# *Chlorella vulgaris* Improves the Regenerative Capacity of Young and Senescent Myoblasts and Promotes Muscle Regeneration

**DOI:** 10.1155/2019/3520789

**Published:** 2019-06-04

**Authors:** Nurhazirah Zainul Azlan, Yasmin Anum Mohd Yusof, Ekram Alias, Suzana Makpol

**Affiliations:** ^1^Department of Biochemistry, Faculty of Medicine, Level 17, Preclinical Building, Universiti Kebangsaan Malaysia Medical Centre, Jalan Yaacob Latif, Bandar Tun Razak, Cheras, 56000 Kuala Lumpur, Malaysia; ^2^Department of Basic Medical Sciences for Nursing, Kulliyyah of Nursing, International Islamic University Malaysia, P. O. Box 141, 25710 Kuantan, Pahang, Malaysia

## Abstract

Sarcopenia is characterized by the loss of muscle mass, strength, and function with ageing. With increasing life expectancy, greater attention has been given to counteracting the effects of sarcopenia on the growing elderly population. *Chlorella vulgaris*, a microscopic, unicellular, green alga with the potential for various pharmaceutical uses, has been widely studied in this context. This study is aimed at determining the effects of *C. vulgaris* on promoting muscle regeneration by evaluating myoblast regenerative capacity *in vitro*. Human skeletal myoblast cells were cultured and underwent serial passaging into young and senescent phases and were then treated with *C. vulgaris*, followed by the induction of differentiation. The ability of *C. vulgaris* to promote myoblast differentiation was analysed through cellular morphology, real-time monitoring, cell proliferation, senescence-associated *β*-galactosidase (SA-*β*-gal) expression, myogenic differentiation, myogenin expression, and cell cycle profiling. The results obtained showed that senescent myoblasts exhibited an enlarged and flattened morphology, with increased SA-*β*-gal expression, reduced myogenic differentiation, decreased expression of myogenin, and an increased percentage of cells in the *G*_0_/*G*_1_ phase. Treatment with *C. vulgaris* resulted in decreased SA-*β*-gal expression and promotion of myogenic differentiation, as observed via an increased fusion index, maturation index, myotube size, and surface area and an increased percentage of cells that stained positive for myogenin. In conclusion, *C. vulgaris* improves the regenerative capacity of young and senescent myoblasts and promotes myoblast differentiation, indicating its potential to promote muscle regeneration.

## 1. Introduction

A decrease in performance of bodily functions is observed with the progression of ageing. Various organs and systems, such as the nervous system, digestive system, and cardiovascular system, are affected by ageing. In the muscular skeletal system, a sequential loss of skeletal muscle mass, strength, and function is observed with increasing age. This condition is known as sarcopenia [1, 2]. Sarcopenia has been described as an age-related decline of muscle mass, function, and strength, with high prevalence after ageing [3]. A longitudinal study revealed muscle mass loss at a rate of 0.64% to 0.70% per year in women and 0.80% to 0.98% per year in men, along with muscle strength loss at a rate of 2.5% to 3% per year in women and 3% to 4% per year in men, in people aged 75 years and older [4].

Although sarcopenia manifests in older people, the causes of this condition are multifactorial and involve changes in the body, such as chronic disease, inflammation, and insulin resistance, in addition to environmental factors like nutritional deficiencies, bed rest, and physical inactivity [1]. An average of 36% and 42% of the female body and male body, respectively, consists of skeletal muscle mass that has the ability to contract or stretch to produce skeletal movement. Skeletal muscle generates heat for the maintenance of body temperature, stores protein reserves, and maintains body posture, while also supporting and protecting soft tissues [2, 5, 6].

The negative effects of sarcopenia include a decrease in the number of motor units and muscle fibre size and an increase in muscle fibre atrophy. However, other factors such as nutrition, hormones, metabolism, immunological conditions, and a sedentary lifestyle can also lead to a decrease in muscle mass and strength. These cause increased abnormal gait, impaired oxidative metabolism, poor glucose regulation, weakness, loss of independence, decreased mobility, falls and fractures, and eventually, morbidity, and mortality [2, 5, 7]. Findings from a previous body composition study demonstrated a marked decrease in skeletal muscle mass, changes in muscle composition, and a greater infiltration of fat into muscles in individuals with sarcopenia, which is associated with ageing [8].

Currently, sarcopenia is an alarming problem in the elderly due to longer life expectancies. Several strategies have been used to fight sarcopenia, such as physical exercise, nutritional supplements, and hormone therapy, e.g., testosterone and oestrogen, which have been shown to improve muscle mass and strength [7, 9, 10]. Greater attention has been given to dealing with the outcomes of sarcopenia, with the aim of reducing the effects of this age-associated disability. In this study, *Chlorella vulgaris* was used to treat myoblast cells in culture in an attempt to determine its effect on the promotion of myoblast differentiation.

*C. vulgaris* was discovered in 1890 by a Dutch researcher named Martinus Willem Beijerinck, who described it as coccoid green algal “balls” with well-defined nuclei [11, 12]. *C. vulgaris* is a microscopic, unicellular freshwater green alga that contains highly nutritious substances such as proteins, nucleic acids, carbohydrates, chlorophylls, vitamins, and minerals and has been widely studied thanks to its potential applications in the pharmaceutical industry [13]. It also contains *β*-carotene, lutein, chlorophyll-a and chlorophyll-b, ascorbic acid, tocopherol, riboflavin, and retinol [14, 15].

Various studies have reported on the beneficial effects of *C. vulgaris*, such as its hypolipidemic action [16, 17] and its effects against diabetes [18, 19] and cancer [20–22]. A previous study reported that glucose and insulin resistance were increased and triglyceride, cholesterol, and free fatty acid levels were decreased in high-fat diet-induced insulin-resistant obese mice treated with *C. vulgaris* [17]. In a liver cancer rat model, treatment with *C. vulgaris* decreased hepatocyte proliferation by decreasing Bcl-2 expression and promoted apoptosis by increasing caspase-8 expression [21]. These potential protective effects of *C. vulgaris* might be due to the presence of bioactive compounds. This study is aimed at determining the effects of *C. vulgaris* on the differentiation of myoblast cells during the formation of mature myotubes in culture and thus investigated its potential for the promotion of muscle regeneration to combat sarcopenia.

## 2. Materials and Methods

### 2.1. Experimental Design

Human skeletal muscle myoblast (HSMM) cells (Lonza, Walkersville, MD, USA) were chosen as a model of replicative senescence in this study. The myoblast cells underwent serial passaging to reach the desired population doubling (PD), and a lifespan curve was determined. The morphology of myoblast cells was observed throughout the serial passaging. A senescence biomarker, SA-*β*-gal, was measured in young and senescent myoblast cells, in addition to myogenic purity, to allow for dependable statistical analysis of different PDs. The viability of control and *C. vulgaris*-treated cells was determined by the CellTiter 96® Aqueous Non-Radioactive Cell Proliferation Assay (MTS; Promega, Madison, WI, USA) and monitored by real-time monitoring using the iCELLigence system (ACEA Biosciences Inc., San Diego, CA, USA). After the optimum dosage of *C. vulgaris* was administered, myoblast cells were induced to differentiate. The differentiation of myoblast cells into mature myotubes was further characterized on days 1, 3, 5, and 7 of differentiation induction by determining the fusion index, maturation index, and myotube size and surface area. The number of cells expressing the differentiation marker myogenin was also determined. This was followed by determination of the cell cycle profile using a fluorescence-activated cell sorter (FACS), the BD FACSVerse^TM^ flow cytometer (Becton Dickinson, USA).

### 2.2. Cell Culture

Human skeletal muscle myoblasts (HSMM) were purchased from Lonza (Walkersville, MD, USA) from two different donors, a 20-year-old Caucasian female and a 17-year-old Caucasian female. The skeletal muscle myoblasts were cultured in skeletal muscle basal medium (SkBM) with supplementation of 50 ml foetal bovine serum (FBS), 10 ml L-glutamine, 0.5 ml human epidermal growth factor (hEGF), 0.5 ml dexamethasone, and 0.5 ml gentamicin/amphotericin-B (Lonza, Walkersville, MD, USA). Cells were cultured at 37°C in a humid atmosphere containing 5% CO_2_. The skeletal muscle myoblast cells then underwent serial passaging to reach senescence. The population doubling (PD) of the cells was calculated for each passage according to the formula ln (*N*/*n*)/ln 2, where *N* is the number of cells at the harvesting stage and *n* is the number of cells at the seeding stage [23]. The starting PD for this study was PD 8. The skeletal muscle myoblast cells reached replicative senescence when the cells could no longer proliferate, as indicated by a very slow proliferation rate even with consecutive replenishment. Morphological changes in the myoblast cells were observed throughout passaging, and the myoblast cell lifespan curve was developed based on the PD and number of days.

### 2.3. Real-Time Monitoring

The iCELLigence system (ACEA Biosciences Inc., San Diego, CA, USA) was utilized to monitor cellular events in real time by recording the electrical impedance signal, followed by converting the impedance value into a cell index (CI) value. The CI is an arbitrary unit that reflects the cell number, morphology, and viability in a given culture well. 1 × 10^4^ myoblast cells were plated in each well of an E-plate L8 and further cultivated at 37°C in a humid atmosphere containing 5% CO_2_. Seeding was allowed for 24 h followed by treatment with *C. vulgaris* and incubation for up to seven days. The CI value was recorded every 10 minutes, and the graph of myoblast cell proliferation was plotted using RTCA Data Analysis Software version 1 (ACEA Biosciences Inc., San Diego, CA, USA). Two E-plate L8 plates were run simultaneously for all dosages of *C. vulgaris* treatment, with two replicates (*n* = 2) for all treatment groups.

### 2.4. Determination of Myogenic Purity

Immunocytochemistry was used to determine myoblast cell purity. Skeletal muscle myoblast cells were seeded at a density of 5 × 10^3^ cells per well in a 96-well plate. After cells were washed with phosphate buffer saline (PBS), the cells were fixed in cold 100% ethanol for 10 minutes followed by incubation with 1% FBS for 30 minutes, with 3x PBS washes in between these procedures. Then, the cells were incubated sequentially with an anti-desmin monoclonal antibody in a dark environment for 1 h (D33, Dako, Glostrup, Denmark) and Alexa Flour 488 goat anti-mouse in a dark environment for 1 h 45 min (Life Technologies, Carlsbad, CA, USA), with 3x PBS washes in between incubations. Hoechst 33342 (Life Technologies, Carlsbad, CA, USA) was then used to visualize the cell nuclei. The cells were viewed under an EVOS FL Digital Inverted Fluorescence Microscope (Life Technologies, Carlsbad, CA, USA). The percentage of desmin-positive cells was determined by examining a minimum of 50 cells from three independent cultures [24].

### 2.5. Determination of Senescence Biomarkers

Identification of senescent skeletal muscle myoblast cells was carried out using the Senescent Cell Histochemical Staining Kit (Sigma-Aldrich, St. Louis, Missouri, USA) according to the manufacturer's instructions. This assay detects the activity of *β*-galactosidase, which is normally present in senescent cells. Briefly, cells at a density of 8 × 10^4^ were cultured in a 6-well plate, washed twice with PBS, and fixed using fixation buffer for 7 minutes. Then, cells were washed with PBS three times before overnight incubation in the staining mixture solution at 37°C in the absence of CO_2_. Blue-stained cells were observed under a light microscope using 40x magnification. The percentage of blue-stained cells versus the total number of counted cells was calculated, with a minimum of 100 cells being observed.

### 2.6. Preparation of *Chlorella vulgaris*

A stock of *C. vulgaris* Beijerinck (strain 072) was obtained from the University of Malaya Algae Culture Collection (UMACC, Malaysia). The stock was grown in Bold's Basal Medium (BBM) with a 12 h dark and 12 h light cycle. The alga was then harvested by centrifugation at 1000 rpm and dried using a freeze dryer. Later, the alga was dissolved in distilled water at a concentration of 10% (*w*/*v*) and boiled at 100°C for 20 minutes using the reflux method. The alga was centrifuged and lyophilised using a freeze dryer to obtain *C. vulgaris* in a powdered form.

### 2.7. Cell Viability Assay

Cell viability was determined using the CellTiter 96® Aqueous Non-Radioactive Cell Proliferation Assay (MTS; Promega, Madison, WI, USA) according to the manufacturer's instructions. A total of 5 × 10^3^ cells was cultured in a 96-well plate and incubated in a CO_2_ incubator at 37°C for 24 h. Then, the media were replaced with media containing *C. vulgaris* at various concentrations—0, 10, 50, 100, 200, 300, 400, and 500 *μ*g/ml—and left in the CO_2_ incubator at 37°C for 24 h. Next, 20 *μ*l of 3-(4,5-dimethylthiazol-2-yl)-5-(3-carboxymethoxyphenyl)-2-(4-sulfophenyl)-2H-tetrazolium/phenazine methosulfate (MTS/PMS) solution was added into each well in a dark environment and cells were further incubated in the CO_2_ incubator at 37°C for 2 h. The absorbance of MTS formazan was measured at 490 nm with a multimode plate reader (PerkinElmer, Waltham, MA, USA). The average reading of control myoblast cells was used to represent 100% cell viability, and the averages of triplicate readings of different concentrations of *C. vulgaris* were converted to a percentage value. The optimum dose of treatment was identified and used for subsequent experiments.

### 2.8. Induction of Myogenic Differentiation

For induction of muscle cell differentiation, the proliferation medium SkBM was replaced with a differentiation medium, DMEM:F12 (Lonza, Walkersville, MD, USA) with supplementation of 2% horse serum (ATCC, Baltimore, USA). The differentiation medium was changed every two days until the desired day of differentiation for parameter measurement.

### 2.9. Determination of Myogenic Differentiation

The differentiation of myoblast cells into mature myotubes was represented using the fusion index, maturation index, and myotube size and surface area, which revealed the efficiency of myogenic differentiation. Myoblast cells were cultured in a 96-well plate, and immunocytochemistry was used to stain the differentiated cells on days 1, 3, 5, and 7 of differentiation as described earlier in the myogenic purity methodology. The myotube surface area was measured using ImageJ software version 1.50i (National Institutes of Health, USA). The myotube size was determined by the number of nuclei per myotube in a minimum of 11 multinucleated cells in 3 different randomly chosen optical fields [24]. The formulas for calculating the fusion index [25] and maturation index [26] are shown below, and a minimum of 50 nuclei was counted in 3 different randomly chosen optical fields.

(1) $$\begin{array}{c} \text{Fusion index}=\frac{\text{the number of nuclei in myotubes}\left(\geq 2\text{ nuclei}\right)}{\text{the total number of desmin}‐\text{positive nuclei}}\times 100\%,\\ \text{Maturation index}=\frac{\text{the number of nuclei in myotubes}\left(\geq 5\text{ nuclei}\right)}{\text{the total number of desmin}‐\text{positive nuclei}}\times 100\%. \end{array}$$

### 2.10. Determination of Myogenin Expression

The number of cells expressing myogenin was determined on day 1 and day 3 of differentiation using immunocytochemistry as described earlier in the myogenic purity methodology, but with a different primary antibody: a mouse monoclonal anti-myogenin antibody (F5D, Dako, Produktionsvej, Denmark) at a 1 : 20 dilution at 4°C overnight. This was followed by incubation with the secondary antibody Alexa Fluor 488 at a 1 : 1000 dilution at room temperature for 1 h 45 min. Cells were incubated with Hoechst 33342 at room temperature for 10 minutes to visualize the nuclei. The cells were viewed under an EVOS FL Digital Inverted Fluorescence Microscope (Life Technologies, Carlsbad, CA, USA). Myogenin expression was calculated as the percentage of cells with myogenin-positive nuclei compared to the total number of nuclei.

### 2.11. Analysis of Cell Cycle by Flow Cytometry

DNA content was determined using the BD Cycletest^TM^ Plus DNA kit (Becton Dickinson, San Jose, CA, USA) according to the manufacturer's instructions. A total of 5 × 10^5^ myoblast cells were washed with buffer solution before staining. The cells were first resuspended in Solution A and incubated at room temperature for 10 minutes. Next, Solution B was added and cells were incubated at room temperature for 10 minutes. Finally, Solution C was added and cells were incubated in the dark at 4°C for 10 minutes. Cells were then filtered with a 35 *μ*m cell strainer cap. The cell cycle phase distribution of nuclear DNA was determined using a flow cytometer fluorescence-activated cell sorter (FACS), the BD FACSVerse^TM^ flow cytometer (Becton Dickinson, USA). A total of 10,000 events were acquired, and the data obtained was analysed using MODFIT software (FACSCalibur BD, USA). The DNA content (*x*-axis, PI fluorescence) versus counts (*y*-axis) was plotted as a histogram.

### 2.12. Statistical Analysis

Data obtained were expressed as means ± SD, and statistical analysis was carried out using SPSS software version 23. Data were analysed using one-way ANOVA followed by Tukey's post hoc test for comparison between treatment and days and between dosages of treatments on a desired day. A *p* value < 0.05 was considered statistically significant.

## 3. Results

### 3.1. Myoblast Cells as an *In Vitro* Model of Cellular Senescence

**Figure 1:**
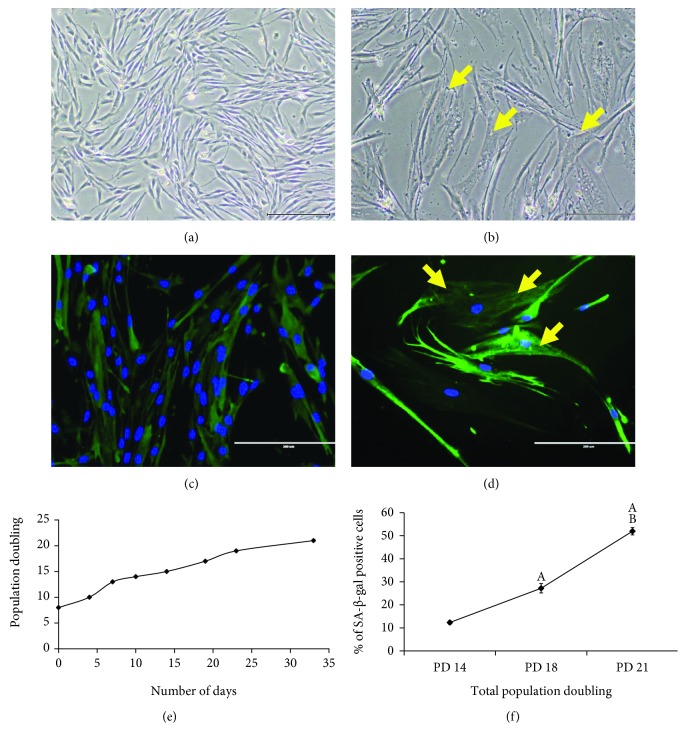
Morphological changes and serial passaging of myoblast cells in culture. Myoblast cells exhibited different morphological characteristics, as seen in the photomicrographs of young (a) and senescent (b) myoblast cells (magnification: 50x), and the photomicrographs of desmin staining of young (c) and senescent (d) cells (magnification: 200x). Myoblasts were stained for desmin (green) and nuclei (blue). Arrows indicate the intermediate filaments and vacuoles observed in senescent myoblast cells. Myoblast cells also lost their proliferative capacity with serial passaging as observed in the proliferation-lifespan curve of myoblast cells (e) and in the increased percentage of cells that stained positive for SA-*β*-gal at higher PDs (f). The data are presented as the means ± SD, *n* = 3. ^A^*p* < 0.05: significantly different compared to myoblasts at PD 14 (young); ^B^*p* < 0.05: significantly different compared to myoblasts at PD 18 (presenescent), with a post hoc Tukey HSD test.

The morphology of myoblast cells at PD 14 (young) and PD 21 (senescent) exhibited different characteristics: cells at PD 14 were spindle shaped with round nuclei, more branching, and multinucleation (Figure 1(a)), while cells at PD 21 were larger and flatter with few nuclei and less branching. Intermediate filaments became more prominent at PD 21, and the presence of vacuoles was observed (Figure 1(b)). Desmin staining was also performed to further elucidate the morphology of myoblast cells at PD 14 (Figure 1(c)) and PD 21 (Figure 1(d)). Myoblast cells at PD 14 were multinucleated, and myoblast cells at PD 21 showed formation of intermediate filaments. Cells at PD 21 also possessed a slower proliferation rate even with consecutive renewal of growth media. Serial passaging was carried out on myoblasts from both donors to achieve replicative senescence. A lifespan curve of myoblasts from the 20-year-old donor was plotted showing cumulative population doublings (PD) versus the number of days (Figure 1(e)). The lifespan curve showed a progressive increase in the proliferation of myoblast cells as the number of days increased. However, the lifespan curve began to plateau at higher PDs, indicating a slower proliferation rate as cells moved towards replicative senescence. The increase in PD resulted in an increased percentage of cells that stained positive for SA-*β*-gal, as seen through the significantly higher SA-*β*-gal expression observed in PD 21 cells compared to both PD 14 and PD 18 cells (Figure 1(f)). Thus, myoblast cells at PD 14 were considered young, while cells above PD 20 were considered senescent cells. The presence of more than 92% desmin-positive cells in each PD of the myoblasts confirmed no loss of myogenicity, allowing for dependable statistical comparison among PDs throughout the study (Table 1).

### 3.2. Effects of *C. vulgaris* on Cell Viability and Proliferation

**Figure 2:**
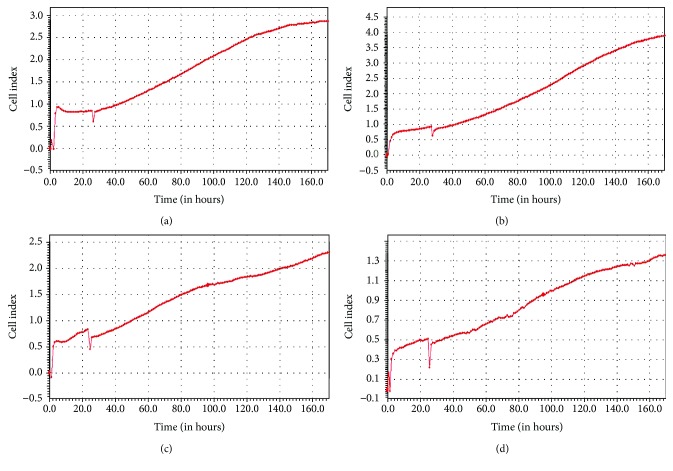
The proliferation rates of myoblast cells from two different donors were represented using cell indexes (CI) for (a) young myoblasts from the 17-year-old donor, (b) senescent myoblasts from the 17-year-old donor, (c) young myoblasts from the 20-year-old donor, and (d) senescent myoblasts from the 20-year-old donor. Data are presented as means, *n* = 2.

**Figure 3:**
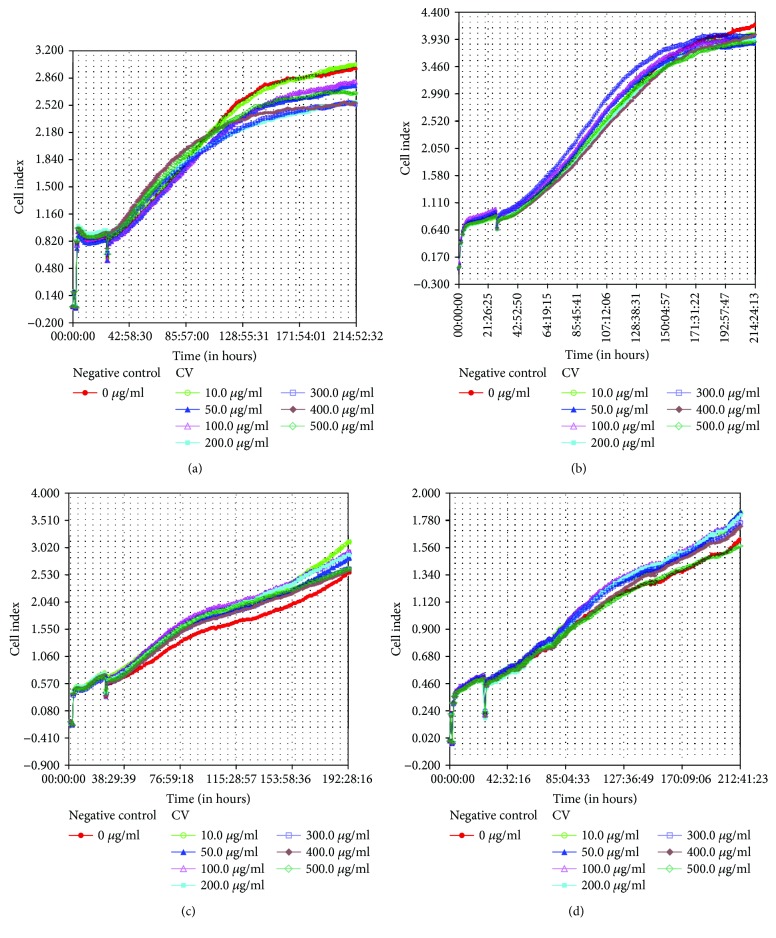
The proliferation rates of myoblast cells from two different donors treated with different concentrations of *C. vulgaris* were calculated using the cell indexes (CI) for (a) young myoblasts from the 17-year-old donor, (b) senescent myoblasts from the 17-year-old donor, (c) young myoblasts from the 20-year-old donor, and (d) senescent myoblasts from the 20-year-old donor. Myoblast cells without *C. vulgaris* treatment is considered as negative control. Data are presented as means, *n* = 2.

Real-time recording by iCELLigence was applied to determine the cell indexes of myoblasts from both donors at PD 14 (young cells) and PD 21 (senescent cells). The cell index graph of both PDs showed an increase in the cell indexes throughout the seven days of incubation for myoblasts from both the 17-year-old (Figures 2(a) and 2(b)) and 20-year-old (Figures 2(c) and 2(d)) donors. Treatment with *C. vulgaris* at various concentrations did not affect the proliferation of myoblast cells at both PDs for both the 17-year-old (Figures 3(a) and 3(b)) and 20-year-old (Figures 3(c) and 3(d)) donors which was increased with increasing number of days. No significant difference was observed in the cell proliferation and viability of myoblasts from both donors when treated with *C. vulgaris* at different PDs. However, myoblast cells from the 20-year-old donor exerted a greater index of proliferation throughout the study when treated with *C. vulgaris* as compared to myoblasts from the 17-year-old donor (Figures 3(c) and 3(d)). Therefore, for subsequent experiments, myoblasts from the 20-year-old donor were used.

**Figure 4:**
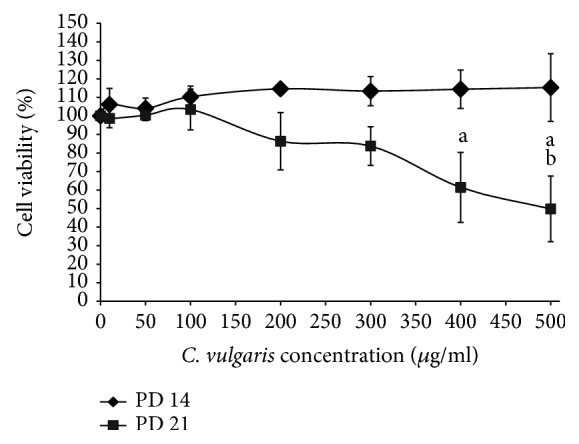
*C. vulgaris* treatment helped to maintain the cell viability of young myoblasts and decrease the viability of senescent myoblasts at high concentrations (400 and 500 *μ*g/ml), as shown in the dose-response curve for *C. vulgaris* treatment in young and senescent myoblast cells. The data are presented as the means ± SD, *n* = 3. ^a^*p* < 0.05: significantly different compared to 0, 10, 50, and 100 *μ*g/ml; ^b^*p* < 0.05: significantly different compared to 200 *μ*g/ml, with a post hoc Tukey HSD test.

The cell viability test demonstrated that incubation with *C. vulgaris* at various concentrations maintained the viability of young (PD 14) myoblast cells. However, a significant decrease in the viability of senescent (PD 21) myoblasts was observed when cells were treated with *C. vulgaris* at concentrations of 400 and 500 *μ*g/ml (Figure 4). Myoblast cells treated with *C. vulgaris* at concentrations of 10 and 100 *μ*g/ml demonstrated the highest percentage of viable cells in both young and senescent myoblasts. Therefore, *C. vulgaris* at concentrations of 10 and 100 *μ*g/ml were chosen for subsequent experiments.

### 3.3. Effects of *C. vulgaris* on the Replicative Senescence of Human Myoblasts

**Figure 5:**
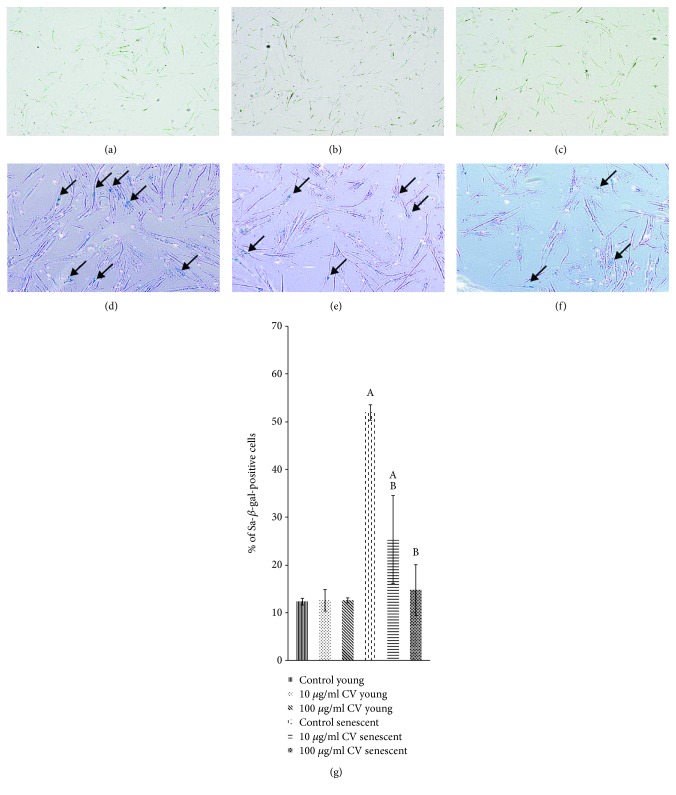
*C. vulgaris* treatment decreased the percentage of SA-*β*-gal-positive senescent cells. SA-*β*-gal staining was used as a senescence biomarker, shown in photomicrographs of young control cells (a), young cells treated with 10 *μ*g/ml *C. vulgaris* (b), young cells treated with 100 *μ*g/ml *C. vulgaris* (c), senescent control cells (d), senescent cells treated with 10 *μ*g/ml *C. vulgaris* (e), and senescent cells treated with 100 *μ*g/ml *C. vulgaris* (f) (magnification: 40x). This data was quantified by the percentage of cells that stained positive for SA-*β*-gal (g). The data are presented as the means ± SD, *n* = 3. ^A^*p* < 0.05: significantly different compared to young controls; ^B^*p* < 0.05: significantly different compared to senescent controls, with a post hoc Tukey HSD test.

Young (PD 14) and senescent (PD 21) myoblasts undergoing various treatments were stained for SA-*β*-gal as shown in Figures 5(a)–5(f). The percentage of cells that stained positive for SA-*β*-gal was significantly higher in senescent myoblasts compared to young cells (Figure 5(g)). However, treatment of senescent cells with *C. vulgaris* at 10 and 100 *μ*g/ml decreased the percentage of cells that stained positive for SA-*β*-gal compared to untreated controls (*p* < 0.05).

### 3.4. Effect of *C.vulgaris* on the Promotion of Myoblast Differentiation

**Figure 6:**
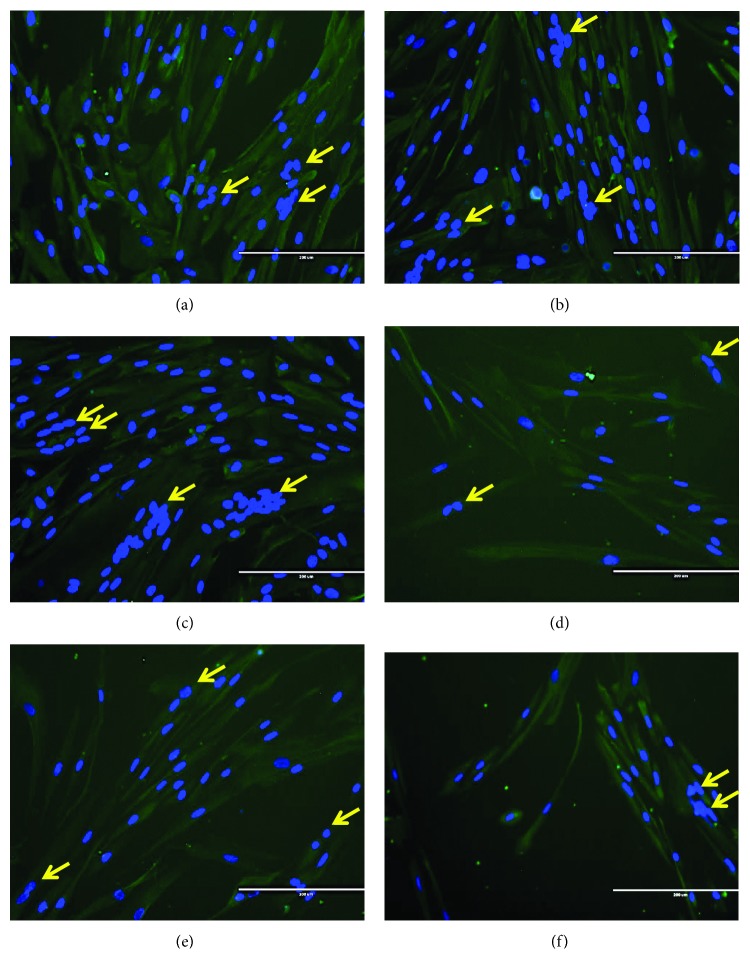
Desmin staining of differentiated myoblasts, indicating the presence of multinucleated cells in mature myotubes. Photomicrographs of desmin staining on day 3 for young control myoblasts (a), young myoblasts treated with 10 *μ*g/ml *C. vulgaris* (b), young myoblasts treated with 100 *μ*g/ml *C. vulgaris* (c), senescent control myoblasts (d), senescent myoblasts treated with 10 *μ*g/ml *C. vulgaris* (e), and senescent myoblasts treated with 100 *μ*g/ml *C. vulgaris* (f) (magnification: 200x). Myoblasts were stained for desmin (green) and nuclei (blue). Arrows indicate the multinucleated cells formed during the differentiation and fusion process.

Young (PD 14) and senescent (PD 21) myoblast cells were differentiated for 7 days to form myotubes. Photomicrographs of desmin staining for young control myoblasts and *C. vulgaris*-treated young myoblasts are shown in Figures 6(a)–6(c), and those for senescent control myoblasts and *C. vulgaris*-treated senescent myoblasts are shown in Figures 6(d)–6(f). Myoblast differentiation was determined by the ability of myoblasts to differentiate, fuse, and form mature multinucleated myotubes. Young control myoblast cells were able to fuse together, forming large, branched, multinucleated myotubes (Figure 6(a)). With *C. vulgaris* treatment, more multinucleated myotubes formed, as shown in Figures 6(b) and 6(c). In senescent control myoblasts, the myotubes formed were smaller with fewer branches compared to those in young myoblasts (Figure 6(d)). However, the formation of myotubes in senescent myoblasts improved with *C. vulgaris* treatment and more branched, multinucleated myotubes were observed (Figures 6(e)–6(f)).

**Figure 7:**
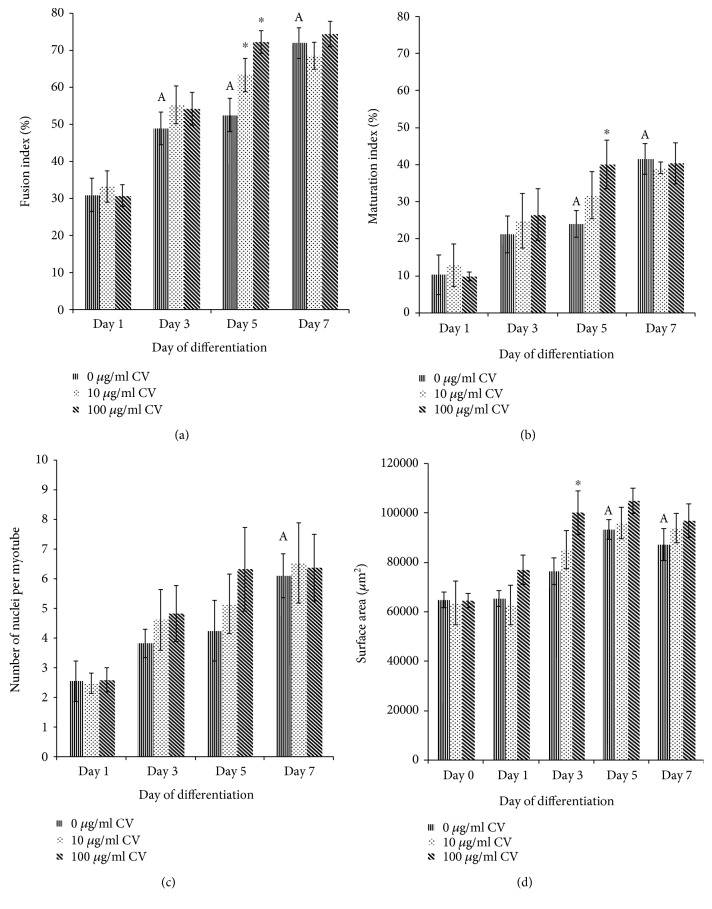
Index of differentiation for young myoblasts. The fusion indexes (a), maturation indexes (b), myotube sizes (c), and myotube surface areas (d) of young myoblasts. Data are presented as means ± SD, *n* = 3. ^∗^*p* < 0.05: significantly different compared to young control myoblasts on a given day; ^A^*p* < 0.05: significantly different compared to young control myoblasts on day 1, with a post hoc Tukey HSD test.

The fusion index of young control myoblasts was significantly higher on days 3, 5, and 7 of differentiation compared to day 1 (*p* < 0.05) (Figure 7(a)). Treatment with *C. vulgaris* at 10 and 100 *μ*g/ml significantly increased the fusion index of young myoblasts compared to young control myoblasts on day 5 of differentiation (*p* < 0.05).

The maturation index of young control myoblasts was significantly higher on days 5 and 7 of differentiation compared to day 1 (*p* < 0.05) (Figure 7(b)). Treatment with *C. vulgaris* at 100 *μ*g/ml significantly increased the fusion index of young myoblasts compared to young control myoblasts on day 5 of differentiation (*p* < 0.05).

The number of nuclei per myotube in young control myoblasts was significantly higher on day 7 of differentiation compared to day 1 (*p* < 0.05) (Figure 7(c)), while the surface area of myotubes of young control myoblasts was significantly higher on days 5 and 7 of differentiation compared to day 1 (*p* < 0.05) (Figure 7(d)). Treatment with *C. vulgaris* at 100 *μ*g/ml significantly increased the surface area of myotubes of young myoblasts compared to young control myoblasts on day 3 of differentiation (*p* < 0.05).

**Figure 8:**
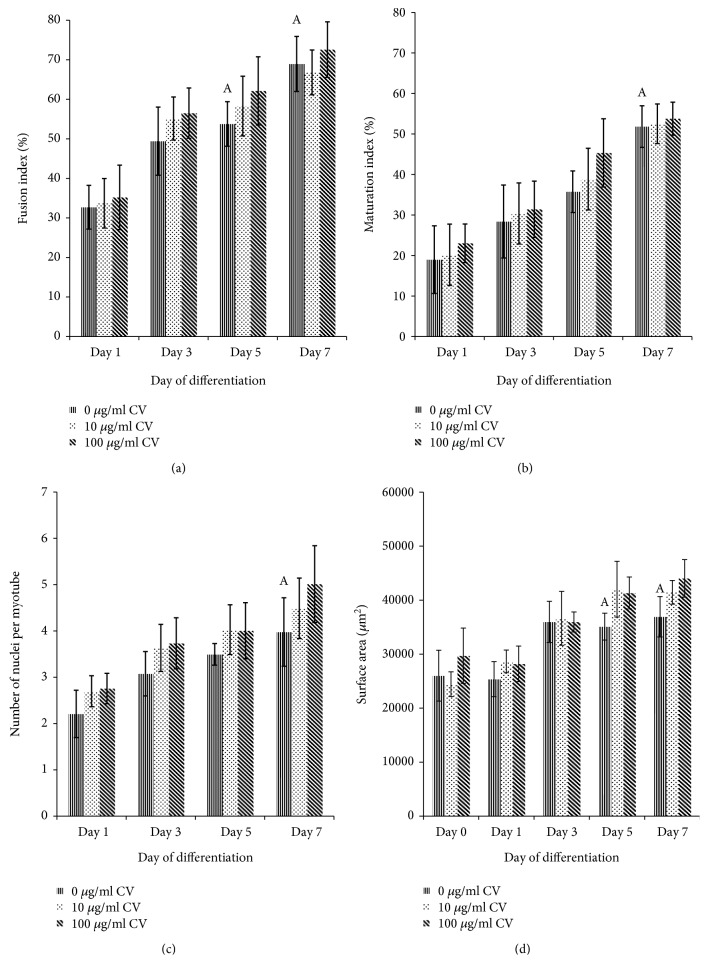
Index of differentiation for senescent myoblasts. The fusion indexes (a), maturation indexes (b), myotube sizes (c), and myotube surface areas (d) of senescent myoblasts. The data are presented as the means ± SD, *n* = 3. ^A^*p* < 0.05: significantly different compared to senescent control myoblasts on day 1, with a post hoc Tukey HSD test.

For senescent myoblast cells, the fusion index and myotube surface area of senescent control myoblasts were significantly increased on days 5 and 7 of differentiation (*p* < 0.05) (Figures 8(a) and 8(d)) while the maturation index and the number of nuclei per myotube were significantly increased on day 7 of differentiation (*p* < 0.05) (Figures 8(b) and 8(c)). No significant differences were observed in the fusion indexes, maturation indexes, number of nuclei per myotube, and myotube surface areas of senescent myoblasts treated with *C. vulgaris* compared to the controls on a given day.

**Figure 9:**
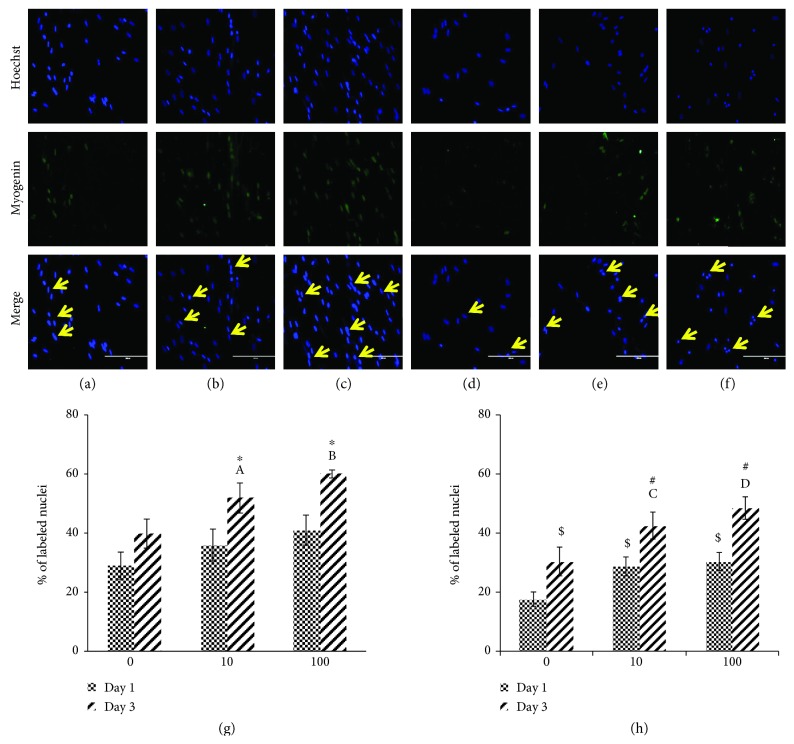
Myogenin expression in young and senescent myoblasts during differentiation induction. Photomicrographs of myogenin staining on day 3 for young control myoblasts (a), young myoblasts treated with 10 *μ*g/ml *C. vulgaris* (b), young myoblasts treated with 100 *μ*g/ml *C. vulgaris* (c), senescent control myoblasts (d), senescent myoblasts treated with 10 *μ*g/ml *C. vulgaris* (e), and senescent myoblasts treated with 100 *μ*g/ml *C. vulgaris* (f) (magnification: 200x), with the presence of nuclei (blue) and myogenin (green) shown using arrows. Quantification of myogenin expression on days 1 and 3 of differentiation for young myoblasts (g) and senescent myoblasts (h). The data are presented as the means ± SD, *n* = 3. ^∗^*p* < 0.05: significantly different compared to young control myoblasts on day 3; ^A^*p* < 0.05: significantly different compared to young myoblasts treated with 10 *μ*g/ml *C. vulgaris* on day 1; ^B^*p* < 0.05: significantly different compared to young myoblasts treated with 100 *μ*g/ml *C. vulgaris* on day 1; ^$^*p* < 0.05: significantly different compared to senescent control myoblasts on day 1; ^#^*p* < 0.05: significantly different compared to senescent control myoblasts on day 3; ^C^*p* < 0.05: significantly different compared to senescent myoblasts treated with 10 *μ*g/ml *C. vulgaris* on day 1; ^D^*p* < 0.05: significantly different compared to senescent myoblasts treated with 100 *μ*g/ml *C. vulgaris* on day 1, with a post hoc Tukey HSD test.

Photomicrographs of myogenin staining are shown in Figures 9(a)–9(c) for young myoblast cells and in Figures 9(d)–9(f) for senescent myoblast cells. Quantitative data for myogenin expression showed that there was a significant increase in myogenin expression on day 3 of differentiation in young myoblasts treated with 10 and 100 *μ*g/ml of *C. vulgaris* compared to young control myoblasts (0 *μ*g/ml *C. vulgaris*) (*p* < 0.05) (Figure 9(g)). The expression of myogenin in myoblasts treated with *C. vulgaris* at 10 and 100 *μ*g/ml was also significantly higher on day 3 of differentiation compared to day 1 (*p* < 0.05).

For senescent myoblasts, treatment with 10 and 100 *μ*g/ml of *C. vulgaris* increased the expression of myogenin significantly on days 1 and 3 of differentiation compared to senescent control myoblasts (0 *μ*g/ml *C. vulgaris*) (*p* < 0.05) (Figure 9(h)). On day 3 of differentiation, the expression of myogenin was significantly increased in both control and *C. vulgaris*-treated myoblasts compared to day 1 (*p* < 0.05).

### 3.5. Effects of *C. vulgaris* on Cell Cycle Profiles

**Figure 10:**
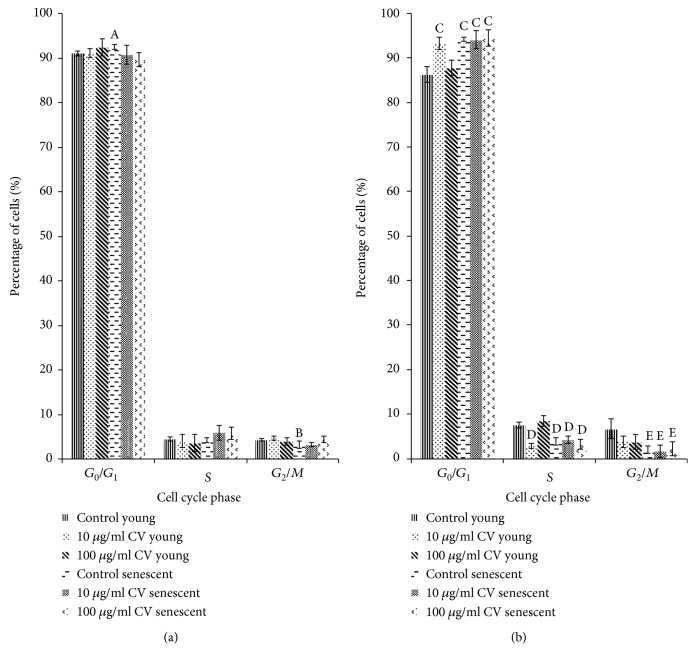
Cell cycle tests were performed on days 0 and 1 of differentiation to determine the myoblast cell population after the induction of differentiation and the effects of treatment with *C. vulgaris*. Cell populations of young and senescent myoblasts both untreated and treated with *C. vulgaris* on (a) day 0 and (b) day 1. The data are presented as means ± SD, *n* = 3. ^A^*p* < 0.05: significantly different compared to young control myoblasts in the *G*_0_/*G*_1_ phase on day 0; ^B^*p* < 0.05: significantly different compared to young control myoblasts in the *G*_2_/*M* phase on day 0; ^C^*p* < 0.05: significantly different compared to young control myoblasts in the *G*_0_/*G*_1_ phase on day 1; ^D^*p* < 0.05: significantly different compared to young control myoblasts in the *S* phase on day 1; ^E^*p* < 0.05: significantly different compared to young control myoblasts in the *G*_2_/*M* phase on day 1, with a post hoc Tukey HSD test.

The percentage of senescent control cells in the *G*_0_/*G*_1_ phase was significantly increased on day 0 of differentiation to 92.45% ± 0.51% compared to that of young control myoblasts at 91.04% ± 0.62%, while the percentage of senescent control cells in the *G*_2_/*M* phase was significantly decreased compared to that of young control cells (Figure 10(a)) (*p* < 0.05). On day 1 of differentiation, young myoblasts treated with 10 *μ*g/ml *C. vulgaris* showed a significant increase in the percentage of *G*_0_/*G*_1_ phase cells and decreased *S* phase cells compared to young control myoblasts (*p* < 0.05) (Figure 10(b)). A similar increase in the percentage of senescent myoblast cells in the *G*_0_/*G*_1_ phase, and decreased *S* phase and *G*_2_/*M* phase cells were observed with *C. vulgaris* treatment on day 1 of differentiation compared to young control myoblasts (*p* < 0.05) (Figure 10(b)).

## 4. Discussion

Although sarcopenia is most common in the elderly, it also affects entire communities due to the economic burden imposed by the disease. Current approaches and interventions for the prevention and management of sarcopenia, such as physical exercise, nutritional supplements, and hormone therapy, show promising results. Higher life expectancies have contributed to an increase in health problems amongst the elderly. Consequently, the introduction of natural remedies such as supplements or dietary interventions has been widely studied. A previous study, which evaluated the effect of *Chlorella* on muscle atrophy in a muscle-specific mitochondrial aldehyde dehydrogenase 2 activity-deficient mouse model, showed that supplementation with *Chlorella* for 6 months resulted in the prevention of age-related muscle atrophy [27].

The current study demonstrated the potential effects of *C. vulgaris* on skeletal myoblast cell regeneration *in vitro*, wherein human skeletal myoblast cells were chosen as a model for replicative senescence. Replicative senescence has been described as an irreversible growth arrest that occurs in cells that have exhausted their proliferative capacity. The results of our study showed that cells undergoing serial passaging *in vitro* achieved a certain number of population doublings (PDs), shown in a constructed lifespan curve. The human skeletal myoblast cells used in this study have the ability to proliferate up to a certain number of proliferation doublings and then lose their capacity to proliferate upon reaching replicative or cellular senescence. Cells were considered young during the first one-third of their lifespan and senescent at the end of their lifespan, after which cells failed to proliferate even with repeated feeding [25].

In the present study, the morphology of myoblast cells at PD 14 exhibited the characteristics of young cells, with more spindle-shaped cells being present. At PD 21, these cells became flatter and larger with the presence of intermediate filaments and vacuoles, indicating senescence, which was also reported by other studies [24, 28]. A previous study reported that the morphology of myoblast cells that reached a senescent state resembled a flattened cell with enlarged cytoplasm and extended cytosolic processes [25]. This could be due to the exhaustion of satellite cells as a large number of degeneration/regeneration cycles occurred [29]. Senescent cells were further verified using SA-*β*-gal staining that stained for *β*-galactosidase activity, which is detectable in senescent cells and undetectable in quiescent cells. Our findings showed that the percentage of cells that stained positive for SA-*β*-gal was significantly higher in PD 20 cells compared to PD 14 and PD 18 myoblasts. Therefore, in this study, myoblasts at PD 14 were used to represent young cells and myoblasts at PD 21 represented senescent cells, which was in line with our previous study [24].

Myogenic purity analysis was carried out to ensure no contamination from myogenic cells, such as fibroblasts, that could obstruct the proliferation of myoblasts. Myogenic purity was maintained in this study, as indicated by the presence of >92% desmin-positive cells in each population doubling. These findings confirmed that there was no loss of myogenicity throughout the replication of experiments and thus that reliable statistical analysis could be carried out between PDs. Real-time recording was performed for seven days on myoblasts from two different donors, showing a similar pattern in the lifespans of myoblasts at both PD 14 and PD 21, as shown by an increase in proliferation along with the increasing number of days.

Various concentrations of *C. vulgaris* were used to treat myoblasts from two different donors, and real-time recording was used to monitor the progression of cells for seven days. An increase in proliferation with the increasing number of days was observed up to a dosage of 500 *μ*g/ml *C. vulgaris*. Previous studies showed that there was no observed difference in myogenic behaviour between myoblasts from young and myoblasts from elderly donors [25, 30]. The dose-response curve of myoblast cells treated with *C. vulgaris* showed increased cell proliferation in young myoblasts treated with up to 500 *μ*g/ml *C. vulgaris* and in senescent myoblasts treated with up to 100 *μ*g/ml *C. vulgaris*. To further elucidate the effects of *C. vulgaris* on replicative senescence, the senescence biomarker SA-*β*-gal was used to identify the presence of senescent cells. The number of cells that stained positive for SA-*β*-gal grew significantly higher with the increase in PD. However, treatment with *C. vulgaris* significantly decreased the activity of SA-*β*-gal in senescent cells, therefore demonstrating the reversal of ageing caused by *C. vulgaris*.

Fusion indexes and maturation indexes were measured to confirm the ability of myoblast cells to differentiate and form mature myotubes, which are multinucleated cells. The findings of this study showed that both young and senescent myoblasts underwent differentiation in culture. However, young myoblasts differentiated more efficiently compared to senescent myoblasts, as indicated by increased fusion indexes, maturation indexes, number of nuclei per myotube, and myotube surface areas on as early as day 3 of differentiation. Treatment with *C. vulgaris* was found to improve the differentiation process in young myoblasts, as shown by increases in the fusion index, the maturation index, and the myotube surface area. However, a similar increase was not observed in senescent myoblasts treated with *C. vulgaris.* These results reveal the ability of *C. vulgaris* to promote cell differentiation and thus myoblast cell regeneration, in young myoblasts.

The differentiation of myoblasts involves the activation of quiescent muscle satellite cells to promote the formation of myotubes through the upregulation of myogenin. Subsequently, immature myotubes are promoted to mature myotubes. Myogenin belongs to a group of muscle-specific regulatory factors (MRFs), proteins that regulate the differentiation of myoblasts [31]. Our results showed that treatment with *C. vulgaris* resulted in a significant increase in myogenin expression on day 3 of differentiation in young myoblasts. In senescent myoblasts, however, myogenin expression was increased on both day 1 and day 3 of differentiation after *C. vulgaris* treatment, indicating the role of *C. vulgaris* in upregulating the expression of myogenin for the promotion of muscle differentiation.

Cell cycle profiles indicate the status of cellular proliferation and the effect of *C. vulgaris* on myoblast differentiation. Our results showed that the percentage of senescent control myoblast cells in the *G*_0_/*G*_1_ phase was significantly increased on day 0 of differentiation, indicating replicative senescence. Cell cycle arrest occurs as a result of the inability of the senescent myoblasts to replicate. On day 1 of differentiation, young myoblast cells treated with 10 *μ*g/ml *C. vulgaris* showed a significant increase in the percentage of *G*_0_/*G*_1_ phase cells and a significant decrease in *S* phase cells, indicating inhibition of cell proliferation and promotion of cell differentiation. A previous study reported that during differentiation, there is inhibition of myoblast proliferation, and thus, more cells are present in the *G*_0_/*G*_1_ phase in order to allow differentiation to take place [32]. However, a similar pattern of cell cycle phases was not observed in senescent myoblasts treated with *C. vulgaris*. The increased percentage of *G*_0_/*G*_1_ phase cells and decreased *S* phase and *G*_2_/*M* phase of senescent cells with *C. vulgaris* treatment were not significant when compared to control senescent myoblasts indicating that these changes were due to the age of senescent myoblasts in culture and not due to *C. vulgaris* treatment. These findings may explain why a more prominent differentiation effect of *C. vulgaris* treatment on young myoblasts was observed in this study.

*C. vulgaris* has been reported on previously due to its potential activity as an antiageing agent by decreasing the expression of aging biomarkers in senescent human diploid fibroblasts (HDFs) [33]. It contains numerous active compounds that contribute to its antiageing properties [34, 35]. However, it may not be possible to identify the specific compound that contributes to this effect. In addition, *C. vulgaris* may produce its beneficial effects through a synergistic response generated by multiple compounds found in these algae [27]. *C. vulgaris* is considered a good source of antioxidants because it contains nutritious substances such as *β*-carotene, lutein, chlorophyll-a, chlorophyll-b, vitamin A (retinol), vitamin B2 (riboflavin), vitamin C (ascorbic acid), vitamin E (tocopherol), and minerals [14, 36, 37].

*C. vulgaris* has also been reported to contain various essential amino acids, including branched-chain amino acids (BCAA) such as valine, leucine, and isoleucine [37–39], which are the vital components of actin- and myosin-composed muscle. Leucine has been reported to be the most potent BCAA for the stimulation of muscle protein synthesis [40–42]. These findings indicate that *C. vulgaris* could be a remedy for the prevention and treatment of sarcopenia. A previous study also reported that the proteins extracted from these microalgae are comparable to or higher than commercial proteins, such as soybean proteins and sodium caseinate [39].

*C. vulgaris* is a good source of n-3 polyunsaturated fatty acids (n-3 PUFAs) due to its high content compared to other microalgae, such as *Spirulina platensis* and *Isochrysis galbana* [15, 43]. A previous study reported that the negative effects of palmitate and tumour necrosis factor-alpha (TNF-*α*) were inhibited by n-3 PUFAs. They also promote differentiation by activating anti-inflammatory pathways within satellite cells [42, 44]. Tocopherol, which is also present in *C. vulgaris*, was shown to help senescent myoblasts to reclaim the morphology of young cells, increasing cell viability and decreasing the expression of SA-*β*-gal [24]. These antioxidant properties of *C. vulgaris* may be responsible for the promotion of myoblast cell differentiation in both young and senescent cells and, thus, the promotion of muscle regeneration, which consequently leads to the reversal of muscle ageing.

## 5. Conclusions

*C. vulgaris* improves the regenerative capacity of young and senescent myoblasts and promotes myoblast differentiation, indicating its potential in promoting muscle regeneration. It may also act as an antiageing agent, as shown by its effects on delaying replicative senescence in myoblast cells.

## Figures and Tables

**Table 1:** The percentage of desmin-positive cells in various population doublings that demonstrated no loss of myogenicity in myoblast cells. The data are presented as the means ± SD, *n* = 3.

Myoblast	PD 14	PD 16	PD 18	PD 20
Desmin positive (%)	97.89 ± 2.12	92.92 ± 1.08	95.55 ± 2.80	94.49 ± 2.54

## Data Availability

The raw data used to support the findings of the study are available from the corresponding author upon request.
